# Supplementary material for: Role of literacy, fear and hesitancy on acceptance of COVID-19 vaccine among village health volunteers in Thailand
Source: PLoS One. 2022 Jun 24;17(6):e0270023. doi: 10.1371/journal.pone.0270023 (PMC9231694; doi:10.1371/journal.pone.0270023)
Supplement: S1 Table — (DOCX) [file pone.0270023.s001.docx]

**S1 Table Effect of literacy, fear and hesitancy vaccine score on acceptance by COVID-19 vaccine types: Univariate and Multivariable analysis**

| **Table A Effect of literacy, fear and hesitancy vaccine score on acceptance of an inactive COVID-19 vaccine** | | | | | |
| --- | --- | --- | --- | --- | --- |
| **Variable** | **Crude OR** | **95%CI** | **Adjusted OR** | **95%CI** | |
| **Vaccine literacy score** | 1.018 | 0.998 - 1.038 | 1.000 | 0.980 - 1.020 | |
| **Vaccine fear score** | 0.927 | 0.896 - 0.958 | 0.933 | 0.901 - 0.966 | |
| **Hesitancy score** | 0.941 | 0.921- 0.961 | 0.937 | 0.912 - 0.963 | |
| **Sex** |  |  |  |  | |
| Male | 1 |  | 1 |  | |
| Female | 0.963 | 0.643- 1.442 | 0.919 | 0.605 - 1.396 | |
| **Age group** |  |  |  |  | |
| <40 | 1 |  | 1 |  | |
| 40-50 | 1.713 | 1.022 - 2.867 | 1.932 | 1.126 - 3.313 | |
| 50-60 | 1.620 | 0.980 - 2.675 | 1.906 | 1.111 - 3.269 | |
| ≥ 60 | 1.007 | 0.551 - 1.837 | 1.150 | 0.598 - 2.213 | |
| **Education** |  |  |  |  | |
| Illiterate | 1 |  | - | - | |
| Elementary School | 0.596 | 0.135 - 2.620 | - | - | |
| High School | 0.888 | 0.203 - 3.686 | - | - | |
| Vocational Certificate | 0.749 | 0.159 - 3.504 | - | - | |
| **Marital status** |  |  |  |  | |
| Single, alone widow | 1 |  | - | - | |
| Couple | 1.003 | 0.706 - 1.423 | - | - | |
| **Religion** |  |  |  |  | |
| Buddhist | 1 |  |  |  | |
| Christ | 0.376 | 0.05 - 2.764 | - | - | |
| Islam | 1.125 | 0.750 - 1.686 | - | - | |
| **Occupational** |  |  |  |  | |
| Agriculture | 1 |  | 1 |  | |
| Own business | 1.001 | 0.622 - 1.609 | 1.089 | 0.669 - 1.772 | |
| Freelancer | 1.711 | 1.196 - 2.445 | 1.818 | 1.253 - 2.637 | |
| Government officer | 2.340 | 0.517 - 10.587 | 2.680 | 0.574 - 12.503 | |
| Private employee | 2.098 | 0.716 - 6.145 | 3.137 | 0.997 – 9.870 | |
| Un hired | 0.931 | 0.494 - 1.7555 | 1.212 | 0.623 - 2.359 | |
| **Comorbidity** |  |  |  |  | |
| No | 1 |  | 1 |  | |
| Diabetes | 0.445 | 0.222 - 0.890 | 0.468 | 0.230 - 0.950 | |
| Hypertension | 1.147 | 0.752 - 1.749 | 1.204 | 0.771 - 1.878 | |
| Hyperlipidemia | 0.501 | 0.180 - 1.390 | 0.532 | 0.188 - 1.499 | |
| Obesity | 0.806 | 0.287 - 2.263 | 0.828 | 0.289 -2.369 | |
| Bone and Skeletal disorder | 1.007 | 0.395 - 2.565 | 1.135 | 0.434 - 2.965 | |
| other | 0.508 | 0.253 - 1.017 | 0.578 | 0.285 - 1.173 | |
| **Income** |  |  |  |  | |
| < 10,000 | 1 |  | - | - | |
| ≥ 10,000 | 1.257 | 0.881 -1.790 | - | - | |
| **Working experienced** |  |  |  |  | |
| <10 | 1 |  | - | - | |
| ≥10 < 20 | 1.041 | 0.721 - 1.502 | - | - | |
| ≥20 | 1.121 | 0.761 - 1.649 | - | - | |
| -: variable did not reach statistical significance in the univariate analysis and not included in the multivariable model | | | | |  |

| **Table B Effect of literacy, fear and hesitancy vaccine score on acceptance of an adenovirus-based COVID-19 vaccine** | | | | |
| --- | --- | --- | --- | --- |
| **Variable** | **Crude OR** | **95%CI** | **Adjusted OR** | **95%CI** |
| **Vaccine literacy score** | 1.017 | 1.006 – 1.028 | 1.010 | 0.994 – 1.018 |
| **Vaccine fear score** | 0.906 | 0.888 – 0.923 | 0.917 | 0.899 – 0.935 |
| **Hesitancy score** | 0.957 | 0.945- 0.968 | 0.947 | 0.933 – 0.961 |
| **Sex** |  |  |  |  |
| Male | 1 |  | 1 |  |
| Female | 1.268 | 0.993- 1.619 | 1.225 | 0.950 – 1.581 |
| **Age group** |  |  |  |  |
| <40 | 1 |  | 1 |  |
| 40-50 | 1.831 | 1.341 – 2.499 | 1.618 | 1.170 – 2.236 |
| 50-60 | 1.647 | 1.214 – 2.232 | 1.445 | 1.044 – 1.999 |
| ≥ 60 | 2.643 | 1.928 – 3.620 | 2.211 | 1.571 – 3.111 |
| **Education** |  |  |  |  |
| Illiterate | 1 |  | - | - |
| Elementary School | 1.496 | 0.438 – 5.098 | - | - |
| High School | 2.166 | 0.637 – 7.363 | - | - |
| Vocational Certificate | 2.652 | 0.764 – 9.197 | - | - |
| **Marital status** |  |  |  |  |
| Single, alone widow | 1 |  | - | - |
| Couple | 0.863 | 0.712 – 1.045 | - | - |
| **Religion** |  |  |  |  |
| Buddhist | 1 |  |  |  |
| Christ | 0.582 | 0.243 – 1.393 | - | - |
| Islam | 1.021 | 0.807 – 1.291 | - | - |
| **Occupational** |  |  |  |  |
| Agriculture | 1 |  | - | - |
| Own business | 0.889 | 0.693 – 1.140 | - | - |
| Freelancer | 0.874 | 0.704 – 1.084 | - | - |
| Government officer | 1.214 | 0.429 – 3.432 | - | - |
| Private employee | 0.435 | 0.151 – 1.245 | - | - |
| Un hired | 1.144 | 0.847 – 1.542 | - | - |
| **Comorbidity** |  |  |  |  |
| No | 1 |  | - | - |
| Diabetes | 0.770 | 0.560 – 1.058 | - | - |
| Hypertension | 1.180 | 0.920 – 1.512 | - | - |
| Hyperlipidemia | 1.084 | 0.706 – 1.664 | - | - |
| Obesity | 0.929 | 0.527 – 1.638 | - | - |
| Bone and Skeletal disorder | 0.929 | 0.527 – 1.638 | - | - |
| other | 0.813 | 0.584 – 1.130 | - | - |
| **Income** |  |  |  |  |
| < 10,000 | 1 |  | - | - |
| ≥ 10,000 | 1.337 | 1.095 -1.631 | 1.147 | 0.920 – 1.414 |
| **Working experienced** |  |  |  |  |
| <10 | 1 |  | - | - |
| ≥10 < 20 | 1.562 | 1.279 – 1.907 | 1.371 | 1.108 – 1.697 |
| ≥20 | 1.259 | 1.005 – 1.576 | 1.006 | 0.784 – 1.293 |
| -: variable did not reach statistical significance in the univariate analysis and not included in the multivariable model | | | | |

| **Table C Effect of literacy, fear and hesitancy vaccine score on acceptance of mRNA-based COVID-19 vaccine** | | | | |
| --- | --- | --- | --- | --- |
| **Variable** | **Crude OR** | **95%CI** | **Adjusted OR** | **95%CI** |
| **Vaccine literacy score** | 0.994 | 0.9795 - 1.007 | 0.984 | 0.969 - 0.998 |
| **Vaccine fear score** | 0.983 | 0.958 - 1.007 | 0.990 | 0.965 - 1.016 |
| **Hesitancy score** | 0.970 | 0.955- 0.985 | 0.963 | 0.945 - 0.982 |
| **Sex** |  |  |  |  |
| Male | 1 |  | 1 |  |
| Female | 1.087 | 0.794- 1.488 | 1.041 | 0.758 - 1.431 |
| **Age group** |  |  |  |  |
| <40 | 1 |  | 1 |  |
| 40-50 | 0.930 | 0.658 - 1.314 | 0.891 | 0.628 - 1.262 |
| 50-60 | 0.974 | 0.701 - 1.351 | 0.965 | 0.693 - 1.342 |
| ≥ 60 | 0.682 | 0.456 - 1.018 | 0.658 | 0.439 - 0.987 |
| **Education** |  |  |  |  |
| Illiterate | NA |  | - | - |
| Elementary School | 1 |  | - | - |
| High School | 1.381 | 1.075 - 1.774 | - | - |
| Vocational Certificate | 1.437 | 0.959 - 2.151 | - | - |
| **Marital status** |  |  |  |  |
| Single, alone widow | 1 |  | - | - |
| Couple | 1.200 | 0.913 - 1.577 | - | - |
| **Religion** |  |  |  |  |
| Buddhist | 1 |  |  |  |
| Christ | 2.096 | 1.049 - 4.184 | - | - |
| Islam | 0.699 | 0.489 - 1.000 | - | - |
| **Occupational** |  |  |  |  |
| Agriculture | 1 |  | - | - |
| Own business | 0.865 | 0.622 - 1.201 | - | - |
| Freelancer | 0.916 | 0.692 - 1.210 | - | - |
| Government officer | NA |  | - | - |
| Private employee | 0.629 | 0.189 - 2.089 | - | - |
| Un hired | 0.621 | 0.380 - 1.012 | - | - |
| **Comorbidity** |  |  |  |  |
| No | 1 |  | - | - |
| Diabetes | 0.824 | 0.540 - 1.255 | - | - |
| Hypertension | 1.001 | 0.704 - 1.422 | - | - |
| Hyperlipidemia | 0.820 | 0.430 - 1.562 | - | - |
| Obesity | 0.719 | 0.305 - 1.691 | - | - |
| Bone and Skeletal disorder | 1.198 | 0.601 - 2.388 | - | - |
| other | 1.342 | 0.925 - 1.947 | - | - |
| **Income** |  |  |  |  |
| < 10,000 | 1 |  | - | - |
| ≥ 10,000 | 1.441 | 1.111 - 1.868 | 1.443 | 1.108 - 1.880 |
| **Working experienced** |  |  |  |  |
| <10 | 1 |  | - | - |
| ≥10 < 20 | 1.135 | 0.867 - 1.484 | - | - |
| ≥20 | 1.002 | 0.742 - 1.352 | - | - |
| -: variable did not reach statistical significance in the univariate analysis and not included in the multivariable model | | | | |

| **Table D Effect of literacy, fear and hesitancy vaccine score on acceptance of cocktailed COVID-19 vaccine** | | | | |
| --- | --- | --- | --- | --- |
| **Variable** | **Crude OR** | **95%CI** | **Adjusted OR** | **95%CI** |
| **Vaccine literacy score** | 1.018 | 1.005 - 1.030 | 1.013 | 1.00 - 1.027 |
| **Vaccine fear score** | 0.992 | 0.972 - 1.012 | 0.993 | 0.972 - 1.014 |
| **Hesitancy score** | 0.956 | 0.943 - 0.968 | 0.986 | 0.971 – 1.002 |
| **Sex** |  |  |  |  |
| Male | 1 |  |  |  |
| Female | 1.332 | 1.017 - 1.742 | 1.268 | 0.962 - 1.672 |
| **Age group** |  |  |  |  |
| <40 | 1 |  | 1 |  |
| 40-50 | 1.404 | 1.057 - 1.863 | 1.238 | 0.923 - 1.661 |
| 50-60 | 1.057 | 0.797 - 1.400 | 0.908 | 0.674 - 1.222 |
| ≥ 60 | 0.709 | 0.502 - 0.999 | 0.574 | 0.398 - 0.828 |
| **Education** |  |  |  |  |
| Illiterate | 1 |  | - | - |
| Elementary School | 1.587 | 0.365 - 6.878 | - | - |
| High School | 2.801 | 0.648 - 12.090 | - | - |
| Vocational Certificate | 3.791 | 0.863 - 16.645 | - | - |
| **Marital status** |  |  |  |  |
| Single, alone widow | 1 |  | - | - |
| Couple | 1.007 | 0.813 - 1.244 | - | - |
| **Religion** |  |  |  |  |
| Buddhist | 1 |  |  |  |
| Christ | 1.637 | 0.884 - 3.029 | 1.627 | 0.864 – 3.306 |
| Islam | 0.443 | 0.316 - 0.618 | 0.434 | 0.307 -0.613 |
| **Occupational** |  |  |  |  |
| Agriculture | 1 |  | - | - |
| Own business | 1.237 | 0.958 - 1.596 | - | - |
| Freelancer | 1.101 | 0.875 - 1.385 | - | - |
| Government officer | 1.643 | 0.580 - 4.654 | - | - |
| Private employee | 0.442 | 0.133- 1.463 | - | - |
| Un hired | 0.959 | 0.671 - 1.369 | - | - |
| **Comorbidity** |  |  |  |  |
| No | 1 |  | - | - |
| Diabetes | 0.638 | 0.443 - 0.918 | 0.695 | 0.478 - 1.011 |
| Hypertension | 0.896 | 0.671 - 1.195 | 1.001 | 0.746 - 1.367 |
| Hyperlipidemia | 1.404 | 0.925 - 2.129 | 1.615 | 1.049 - 2.485 |
| Obesity | 0.547 | 0.258 - 1.158 | 0.546 | 0.254 - 1.176 |
| Bone and Skeletal disorder | 0.752 | 0.389 - 1.451 | 0.993 | 0.505 - 1.954 |
| other | 1.034 | 0.747 - 1.429 | 1.133 | 0.812 - 1.583 |
| **Income** |  |  |  |  |
| < 10,000 | 1 |  | - | - |
| ≥ 10,000 | 1.877 | 1.530 - 2.300 | 1.684 | 1.364 - 2.079 |
| **Working experienced** |  |  |  |  |
| <10 | 1 |  | - | - |
| ≥10 < 20 | 1.183 | 0.954 - 1.465 | - | - |
| ≥20 | 0.881 | 0.686 - 1.131 | - | - |
| -: variable did not reach statistical significance in the univariate analysis and not included in the multivariable model | | | | |
