# Supplementary material for: Role of literacy, fear and hesitancy on acceptance of COVID-19 vaccine among village health volunteers in Thailand
Source: PLoS One. 2022 Jun 24;17(6):e0270023. doi: 10.1371/journal.pone.0270023 (PMC9231694; doi:10.1371/journal.pone.0270023)
Supplement: Attachment — Submitted filename: Response to reviewer.docx [file pone.0270023.s003.docx]

**Response to reviewer**

**Title:** Role of Literacy, Fear, Hesitancy on Acceptance of COVID-19 Vaccine among Health Village Volunteer in Thailand (PONE-D-22-04125)

Thank you very much for your fruitful comments. We have revised the manuscript following your suggestions and comments. We hope our revision would satisfy your expectation. Please find our point-by-point response below.

**Comments to the Author**

Reviewer #1: The authors use cross-sectional data collected from a large and diverse sample of Thai village health volunteers to determine what factors are associated with COVID vaccination intentions. Results indicate that vaccine hesitance and fear are both associated with lower likelihood of getting vaccinated. The theoretical contribution is small, but there is considerable value in studying health behaviors in non-WEIRD (western, educated, industrialized, rich and democratic) countries. However, the manuscript has important shortcomings. There are data limitations, analytic errors, and the writing is unacceptable (e.g., there are numerous grammatical errors). These problems appear to be fixable, though doing so will require considerable effort.
Given the numerous opportunities for improvement, I organize my review in the order in which I first observed each problem.

**1. The introduction is unfocused and often off topic. This manuscript promises to make a modest empirical contribution, documenting attitudes associated with vaccine acceptance in Thailand. The introduction should explain this succinctly, summarizing what is known about other part of the world and explaining why readers might expect Thailand to be different.**

**Ans:** We have re-written the whole introduction following your suggestions. (Page 3, line 51-91)

**2. The description of the sample (page 4 line 100-) and of the procedures for collecting data (page 6 line 163-) do not appear entirely consistent. For example, the former implies that participants were registered users of a mobile app called “SMART VHV.” The latter says that participants were recruited using a form of snowball sampling via social media (Facebook and Line). I encourage the authors to combine these two sections and to carefully edit the content for clarity and consistency.**

**Ans:** We totally agreed. We have combined the two sections. We first described about the registered SMART VHV, for these user group use social media (Line and Facebook). After that we described how we inform and distribute our online questionnaire. (page 4, line: 91-112)

**3. There are important details missing from the description of the sampling. For example, how many individuals were invited to participate? See the AAPOR guidelines for reporting survey data.**

**Ans:** We have revised the manuscript stated that “A nonprobability sampling so called the snowball sampling was adopted based on the 137,782 registered SMART VHV in our study through the social platform (Line and Facebook) in that these two social media are the most popular in Thailand and numbers of VHV use it to communicate and coordinate with each other.” (Page 4, line: 100-102)

**4. The authors imply that the sample is diverse and largely unbiased, but descriptive feature (page 7 line 195) suggest that it is highly skews (e.g., 84% female)**

**Ans:** This research is online data collection, and we did not specify the respondent. We understand that it will cause the data to be skewed. We therefore consider it a limitation of the present studies. (page 15, line 318-323). Moreover, Thai society men need to be the main income earner of the family and have to work outside while women are at home or working at home. Therefore, it is more likely that women will apply for VHV, which is obliged to perform community health care duties, which work mostly during the day.

**5. Figure 1 is missing**

**Ans:** We have submitted the Fig 1 (separate file).

**6. The description of the measures (starting on page 5) appears to contain many errors. For example, how can a 12-item scale include items numbered 11-15? And how can the same item numbers be used in several scales?**

**Ans:** We sincerely apologize for misunderstood in the use of characters. We explain that we planned to utilize the letter l (lowercase l) for the questions in the Vaccine Literacy (VL), which resembles the number 1. So, for the questions in this category, we've changed it to capital L for clarification. (page 5, line 127, 129 and 131-132).

**7.When describing a scale, the authors should also report the M, SD, and alpha.**
 **Ans:** Thank you for your suggestions, we have reported the mean, SD and alpha as written in page 8 line 184-187. “Based on the present study, the mean score and standard deviation (SD) of VL was 33.83 (SD: 7.55), VF was 11.35 (SD: 4.58), and VH was 24.72 (SD:7.45). Cronbach’alpha coeffiecnt for VL, VH and VF were 0.89, 0.91 and 0.90, respectively.” (Page 6, line: 164-166)

**8. What the authors describe as vaccine literacy appears to tap several different ideas, include efficacy. If this is an established scale, a cite would help.**

**Ans:** The VL questionnaire was adapted from Vaccine Literacy Questionnaire-Italy (HlVa-IT) of Biasio LR. et al. (2020). We have added this reference in the Methods section. (Page 5, line 125-126)

**9. What the authors describe as vaccine hesitancy includes questions about effectiveness and perceived importance. If this is an established scale, a cite would help.**

**Ans:** The VH questionnaire was adapted from Hesitancy Scale (aVHS) of Akel HT. et al. (2021). We have added this reference in the Methods section. (Page 6 line: 144-145)

**10. The authors reject the use of a random effect based on the ICC (page 7 line 189). They should report the ICC.**

**Ans:** We have reported the ICC which was 0.053570. (page 7, line 186)

**11. In the text, the authors say that participants were asked to specify their preferred vaccine if they indicated willingness to be vaccinated (page 6 line 148). However, Table 1 reports vaccine preference among those who do not wish to be vaccinated.**

**Ans:** We have noted and corrected our mistaken of reporting number in table 1. (Page 9)

PS: we also re-checked our results and have corrected the number report in Table 2 and Table 3 of adjusted OR.

**12. The occupation categories in Table 1 are not mutually exclusive (e.g., employee and private employee).**

**Ans:** Thank you very much for your comments. We have changed the word from employee to freelancer in all Tables.

**13. If the authors want to demonstrate that the association between vaccine fear and acceptance is conditioned on the individual’s vaccine preference, it would be more appropriate to test** **whether preference moderates the influence of fear when estimating acceptance.**

**Ans:** Thank you very much for your idea about testing of whether preference moderates the influence of fear when estimating acceptance. We did try to analyze but it is impossible to do so, since our questionnaire design was that for those who only chose accepted to get vaccine can later choose the preference types. Therefore, it cannot take the preference type as a covariate in the logistic model.

|  | Accept to get shot | |
| --- | --- | --- |
| Preference type of vaccine | No | Yes |
| No | 2,200 | 0 |
| Inactivated | 0 | 503 |
| Adenovirus | 0 | 665 |
| mRNA | 0 | 1,759 |
| Cocktail | 0 | 59 |
| Any | 0 | 126 |
| Total | 2,200 | 3,112 |

As a result, we decided to analyze the association between VL, VF, VH, and AV in different model based on each preference type of vaccine, adjusted by other variables. We have showed in our univariate, the effect of fear itself on acceptance by vaccine types were not different according to the Odd ratio in the univariate analysis.

**14 The discussion is too long and covers too many topics (including several that we not mentioned in the results section). As with the introduction, the manuscript would be more compelling if the authors restricted their comments to their most important contributions.**
 **Ans:** Thank you very much for your suggestions. We have revised our discussion follow our main aim and results. We arranged the discussion to follow first the result of factors associated with overall acceptance (Table 2) then factors associated with preferences types of vaccine (Table 3). Implication limitation and conclusion. (Page 12-15, line 234-330)

=== Potential PLOS policy violations ===

**The authors checked the box indicating that the data will publicly available, but then write, “The datasets used and /or analyzed during the current study are available from the corresponding author on reasonable request.”**

**Ans:** All relevant data are within the paper and its Supporting Information files.

**Participants were not allowed to submit the questionnaire if they skipped questions (page 7 line 175). Every US-based IRB that I have worked with requires that participants be allowed to refuse to answer questions.**

**Ans:** We would like to extend our meaning of the sentence “Participants were not allowed to submit the questionnaire if they skipped questions” and make it clearer that is participants can allow to refuse to answer the question, or don’t participate, or stop doing the questionnaire at any time. There is a function of google form that you need to answer all question before submitting. We select this function, for we can protect an incomplete missing data. However, there are still have some variable or questions (We did not set up the function) that were incomplete as show in Figure 1.
