# Supplementary material for: Role of literacy, fear and hesitancy on acceptance of COVID-19 vaccine among village health volunteers in Thailand
Source: PLoS One. 2022 Jun 24;17(6):e0270023. doi: 10.1371/journal.pone.0270023 (PMC9231694; doi:10.1371/journal.pone.0270023)
Supplement: Attachment — Submitted filename: Reponse to Reviewers.docx [file pone.0270023.s004.docx]

**Title:** Role of Literacy, Fear, Hesitancy on Acceptance of COVID-19 Vaccine among Health Village Volunteer in Thailand (PONE-D-22-04125)

Thank you very much for your fruitful comments. We have revised the manuscript following your suggestions and comments. We hope our revision would satisfy your expectation. Please find our point-by-point response below.

**Review Comments to the Author**

**Reviewer #2**: The authors have done a thorough revision of the manuscript based on previous comments. However, there are some parts still need to be revised further to make the manuscript better. Please find below a few comments for your consideration.

**Introduction**
**1. Authors presented the vaccination coverage in Thailand as of October 2021. It would be better if the figures for the current vaccination coverage in the country is presented.**

**Ans:** We have stated the current vaccination coverage in May 2022 is 70.3%. (Page 3; line: 58-59)

**2. In the introduction, the authors presented the COVID-19 acceptance rates around the world. To make this information complete, the status in Africa could be added. For instance:** <https://journals.plos.org/plosone/article?id=10.1371/journal.pone.0260575>

**Ans:** We have added the recommended reference as in the sentence “Vaccine acceptance rate was 37.40% in Jordan, 61.16% in Bangladesh, 56.90% in the EU, 80.00% in the USA and 63% in Africa [10-14]”. (Page 3; line: 62)

**3. The introduction still needs to be revised further for grammatical and presentation errors.
Authors should include the month in 2021 when the first batch of the COVID-19 vaccines arrived Thailand.**

**Ans:** The first COVID-19 vaccine arrived in Thailand in February 2021 and the Thai Prime Minister encouraged VHVs to boost public confidence in this vaccine. Later, novel variants of COVID-19 and new vaccines arrived in Thailand (Page 4; line: 81)

**Methods**
**4. “Questionnaires” should be rewritten as “questionnaire” all through the manuscript.**

**Ans:** We have rewritten from questionnaires to questionnaire.

**5. The authors have not stated the exclusion criteria used in the selection of the participants.**

**Ans:** SMART VHVs who lack of address information and incomplete filling questionnaire were excluded. (Page 4-5; line 110-111)

**6. Authors should provide the citations/references of the extensive literature used in this study.**

**Ans:** We have provided citations/references of literature as recommendation.

**7. Authors did not explain how the literacy, fear, and hesitancy scores were calculated before being used in the univariate and regression analysis. Ideally, this explanation should appear under the statistical analysis section.**

**Ans:** VL, VF, and VH score were summed up individually and treated in models as a continuous independent variable. (Page 7, line: 181-182)

**Results**
**8. In Table 1, the heading of the first row should be: Vaccinated n (%) Unvaccinated n (%)
Ans:** We have revised accordingly.

**Discussion**
Has been improved upon.

**Reviewer #3**: Paper by Dr. Wungrath et al. treated social and psychological factors associated with acceptance of COVID-19 vaccine in Thailand. I would like to present comments to improve the manuscript.

**[Major]**
**1. Introduction section may be relatively lengthy. To introduce readers to Methods and Results, shorter introduction may be effective.**

**Ans:** We shorten the introduction part as recommend.

**2. As a reader, I would like the authors to explain vaccine literacy score, vaccine fear score, and vaccine hesitancy score, briefly.**

**Ans:** We have briefly explained all scores as mentioned in the methods part. (Page 5-6; line: 124-150)

**3. Table 3: I am interested in the results of pooled analysis of varied types of vaccines.**

**Ans:** The pooled analysis result (all type of vaccines) have been shown in Table 2.

**4. Table 3: Showing the results of the results of non-adjusted analysis may increase the understanding of this study results.**

**Ans:** Thank you very much for your suggestions. We totally agree. However, we also concern about containing too many tables. Therefore, we put the overall results, showing both non-adjusted and adjusted results in the manuscript (Table 2). We provide the non-adjusted and adjusted full results varied by vaccine types in supporting information (S1 Table).

**[Minor]
5. Many** **abbreviations may interrupt readers to smoothly read the manuscript. Key abbreviations could be spelled out.**

**Ans:** We have reduced some abbreviations. However, according to the journal regulation about abbreviations stated that “Do not use non-standard abbreviations unless they appear at least three times in the text”. Therefore, we keep other key abbreviations in the manuscript.
